# Hospital Cost Components and Predictors in *Escherichia coli* Bacteremia

**DOI:** 10.3390/tropicalmed11050116

**Published:** 2026-04-28

**Authors:** Tri Pudy Asmarawati, Fikri Sasongko Widyatama, Hari Basuki Notobroto, Erwin Astha Triyono, Nasronudin Nasronudin, Motoyuki Sugai, Kuntaman Kuntaman

**Affiliations:** 1 Doctoral Programme of Medical Science, Faculty of Medicine, Universitas Airlangga, Surabaya 60131, Indonesia; tpasmarawati@fk.unair.ac.id; 2 Department of Internal Medicine, Faculty of Medicine, Universitas Airlangga, Surabaya 60131, Indonesia; erwin.astha@fk.unair.ac.id; 3 Department of Internal Medicine, Universitas Airlangga Hospital, Surabaya 60115, Indonesia; 4 Department of Medical Microbiology and Parasitology, Faculty of Medicine, Universitas Airlangga, Surabaya 60131, Indonesia; fikri.sasongko@fk.unair.ac.id; 5 Division of Biostatistics and Population Studies, Faculty of Public Health, Universitas Airlangga, Surabaya 60115, Indonesia; haribasuki.n@fkm.unair.ac.id; 6 Department of Internal Medicine, Dr. Soetomo General Academic Hospital, Surabaya 60286, Indonesia; 7 Institute Tropical of Diseases, Universitas Airlangga, Surabaya 60115, Indonesia; 8 AMR Research Center, National Institute of Infectious Diseases, Japan Institute for Health Security, Tokyo 102-0071, Japan; sugai@niid.go.jp; 9 Department of Antimicrobial Resistance, Hiroshima University Graduate School of Biomedical Science, Hiroshima 739-0046, Japan; 10 Department of Clinical Microbiology, Dr. Soetomo General Academic Hospital, Surabaya 60286, Indonesia

**Keywords:** ESBL-producing *Escherichia coli*, healthcare cost, infectious diseases, antimicrobial resistance, antimicrobial stewardship

## Abstract

**Background/Objectives:** *Escherichia coli* bacteremia is a major cause of morbidity, mortality, and healthcare expenditure. The increasing prevalence of extended-spectrum beta-lactamase (ESBL)-producing *Escherichia coli* (*E. coli*) complicates management and resource utilization. This study aimed to identify clinical predictors of higher hospital costs in *E. coli* bacteremia. **Methods**: We conducted a cross-sectional study of hospitalized patients with *E. coli* bacteremia in Surabaya, Indonesia (2022–2024). Hospital costs were categorized into bed costs, diagnostic costs, pharmacy costs, antibiotic costs, total costs, and daily costs. Costs were compared between ESBL and non-ESBL cases. Predictors of higher hospital costs were analyzed using generalized linear models with a Gamma distribution and log-link. **Results**: Among 209 patients, 131 (62.7%) had ESBL-producing *E. coli*. ESBL *E. coli* bacteremia was associated with significantly higher bed, diagnostic, pharmacy, total, and daily hospital costs than non-ESBL cases, while antibiotic costs were similar. ESBL *E. coli* bacteremia was associated with higher diagnostic and daily costs. High-care/ICU stay was the strongest predictor of increased costs. Pneumonia and infection source influenced cost components. Longer hospitalization increased total cost but reduced daily cost. **Conclusions**: Hospital costs in *Escherichia coli* bacteremia are driven by antimicrobial resistance, disease severity, and healthcare utilization. Targeted strategies such as antimicrobial stewardship and optimized critical care use are essential to reduce the economic burden.

## 1. Introduction

*Escherichia coli* bacteremia remains one of the most common causes of bloodstream infection (BSI) worldwide and is associated with substantial morbidity, mortality, and healthcare expenditure [1,2]. The increasing prevalence of antimicrobial-resistant *E. coli*, particularly ESBL-producing strains, has further complicated management by limiting effective treatment options and increasing resource utilization [3,4,5]. These challenges have important implications not only for patient outcomes but also for hospital costs and health system sustainability [6].

Previous studies have demonstrated that BSIs due to resistant organisms are associated with prolonged hospitalization, increased use of broad-spectrum antimicrobials, and higher overall costs [7,8,9]. However, the extent to which antimicrobial resistance (AMR) itself drives hospital costs, rather than downstream factors such as disease severity, care intensity, and HAI status, remains unclear [10]. It is essential to determine these associations to inform antimicrobial stewardship programs and value-based strategies [11].

Antimicrobial-resistant infections substantially increase hospital spending, but the pattern of extra cost is complex [12]. Cost analyses that break down total expenditure into components such as bed-days, diagnostics, pharmaceuticals, and consumables provide deeper insight into how resistant infections affect healthcare spending, in particular [7]. Understanding whether ESBL-producing *E. coli* bacteremia independently increases costs after adjustment for clinical complexity may help prioritize interventions targeting modifiable cost drivers, including early source control, appropriate antimicrobial selection, and timely de-escalation [13,14].

Therefore, this study aimed to identify clinical predictors of higher hospital costs among patients with *E. coli* bacteremia, with a specific focus on the economic impact of ESBL-producing *E. coli*. We assessed factors associated with several cost components to improve antimicrobial stewardship and cost-containment strategies in the era of rising antimicrobial resistance. 

## 2. Materials and Methods

### 2.1. Study Design, Patient Selection, and Variables Definition

We conducted a cross-sectional study among hospitalized patients with bacteremia caused by *E. coli* in Dr. Soetomo General Academic Hospital, Surabaya, Indonesia. Patients were identified from the blood culture records of the Department of Clinical Microbiology between 2022 and 2024. We included patients aged over 18 years with a positive blood culture for *E. coli* and signs of infection. The exclusion criteria were zero or incomplete hospital cost records and a length of stay (LOS) of less than 1 day. Data on demography and clinical setting were obtained from electronic medical records. Data on hospital costs were obtained from the Department of Accounting and Finance billing records.

Healthcare-associated infection was defined according to the National Healthcare Safety Network (NHSN) definition, in which a positive blood culture revealing *E. coli* was recorded on or after the third day of admission, with day of admission defined as calendar day 1 [15]. The time before bacteremia was defined as the period between hospital admission and the date of positive blood culture. The time after bacteremia was defined as the period between the diagnosis of bacteremia and hospital discharge. The comorbidities were calculated using the Charlson comorbidity index (CCI) and included several comorbid conditions, including myocardial infarction, peripheral vascular disease, cerebrovascular disease, dementia, chronic pulmonary disease, rheumatic disease, peptic ulcer, hemiplegia, liver disease, chronic kidney disease, leukemia, lymphoma, HIV/AIDS, solid tumors, and diabetes mellitus [16]. The serum albumin level, Pitt bacteremia score (PBS), and the sequential organ failure assessment (SOFA) score were recorded at the bacteremia diagnosis. Mortality was defined as all-cause in-hospital death.

### 2.2. Cost Calculation

Hospital costs were divided into four groups: bed costs, diagnostic costs, pharmacy costs, and antibiotic costs. The total cost was the sum of all cost components guaranteed by the National Health Insurance. The bed cost included medical and surgical procedures, doctor, nurse, and expert consultation fees, rehabilitation services, hospital administration fees, and daily room and ICU services. Antibiotic costs were obtained from the antibiotic spending in the Pharmacy department. Pharmacy costs included expenses for non-antibiotic medications, chemotherapy drugs, intravenous fluids, blood products for transfusions, consumables, mechanical ventilation, oxygen therapy, and other pharmaceutical services. Diagnostic costs consisted of radiology, laboratory, and other medical support services. The daily cost was calculated by dividing the total cost by the LOS. The fee was expressed in Rupiah (Rp) and converted to United States Dollar (USD) using the 2023 exchange rate (USD 1 = 15,567.50 IDR) [17].

### 2.3. Statistical Analysis

#### 2.3.1. Descriptive and Bivariate Analysis

We analyzed the data using SPSS version 24 (Chicago, IL, USA; RRID: SCR_002865). Numeric data of demographic and characteristic data were presented as median (IQR), while the categorical data were presented as frequencies and percentage distributions. We categorized characteristics and hospital costs by ESBL status. We assessed the normality of the continuous data using the one-sample Kolmogorov–Smirnov test and obtained a *p*-value < 0.001. A comparison between ESBL and non-ESBL *E. coli* groups was carried out using the Mann–Whitney test for numeric data. Categorical variables were examined with the Chi-square test. Significance was set at *p* < 0.05, with a 95% confidence interval. Missing data were assessed using SPSS Missing Value Analysis. Less than 2.9% of values were missing per variable, and Little’s MCAR test was non-significant (χ^2^ = 2.619, df = 12, *p* = 0.998), suggesting data were missing completely at random. Therefore, listwise deletion was applied.

#### 2.3.2. Predictor of Higher Cost in *E. coli* Bacteremia and ESBL-Producing *E. coli* Bacteremia

We identified independent predictors of higher hospital cost using a generalized linear model (GLM) for skewed cost data. We used a Gamma distribution with a log-link for right-skewed data and compared model performance using the Akaike Information Criterion (AIC) and the Bayesian Information Criterion (BIC). Predictors included in the analysis were selected based on *p* < 0.1 results from bivariate analysis of low-medium and medium-high cost (Tables S1 and S2). Multicollinearity was assessed using variance inflation factors (VIFs) and the tolerance test. Length of stay had perfect collinearity with time before/after bacteremia; therefore, it was excluded from the analysis. To reduce the risk of overfitting, the number of predictors was limited relative to the sample size, and maintained the event per variable (EPV) >15. Comorbidities were represented by CCI, due to the small number of events in some diseases. Mechanical ventilation was excluded due to potential collinearity with other disease severity indices (high-care/ICU and PBS). HAIs were also excluded because we used a definition that was consistent with LOS, time after/time before bacteremia. Other variables with tolerance values of ≥0.27 and VIFs of ≤3.72 were included in the analysis. Predictors included in the final model for the *E. coli* bacteremia cost predictor were sex, ESBL *E. coli*, time before bacteremia, high-care/ICU stay, CCI, pneumonia, urinary tract infection (UTI), intracranial infection, skin/soft tissue infection, inappropriate initial antibiotic therapy (IIAT), and PBS. Predictors included in the final model of ESBL-producing *E. coli* bacteremia cost predictors were sex, time after bacteremia, pneumonia, skin/soft tissue infection, high-care/ICU stay, CCI, and PBS. Predictors with a significant *p*-value (<0.05) were reported in the results. Time before bacteremia was included in the overall *E. coli* bacteremia cost model to account for baseline hospitalization duration prior to infection onset, whereas, for the ESBL *E. coli* bacteremia analysis, time after bacteremia was used to capture additional resource utilization related to the management of infections, which contributes to higher costs.

Sensitivity analyses were performed using alternate model specifications (inverse Gaussian distribution with a log-link and linear regression) and exclusion of extreme cost values.

## 3. Results

### 3.1. Baseline Characteristics

We analyzed 209 patients with *E. coli* bacteremia, and 131 (62.7%) were ESBL-producing *E. coli* (Table 1). The median LOS was 7 (3–14) days and did not differ significantly between the two groups. Comorbidities and CCI did not show differences between groups. Pneumonia was the most common source of bacteremia (*n* = 144, 68.9%). Urinary tract and skin infections were predominantly caused by ESBL-producing *E. coli*, whereas intracranial and intra-abdominal infections were otherwise. Healthcare-associated infections (HAIs) occurred more frequently in patients with ESBL *E. coli* bacteremia. Patients with ESBL infection were more likely to receive IIAT (*n* = 109, 83.2%). The ESBL *E. coli* cases had a higher median PBS (2 vs. 0, *p* < 0.001) and were more likely to require vasopressor use, whereas the SOFA score did not differ significantly. Mortality occurred in 137 (65.6%) of the patients.

### 3.2. Analysis of Hospital Costs Components in E. coli Bacteremia

Patients with ESBL *E. coli* bacteremia had substantially higher hospital costs than those with non-ESBL *E. coli* bacteremia (Table 2). The median costs for bed (871.48 vs. 523.14, *p* = 0.009), diagnostics (613.55 vs. 408.21, *p* < 0.001), pharmacy (869.27 vs. 483.26, *p* = 0.013), total (2,448.15 vs. 1,459.16, *p* = 0.001), and daily expenses (311.73 vs. 252.88, *p* = 0.021) were significantly elevated in the ESBL group. Nonetheless, antibiotic expenditures did not differ significantly across groups. The findings indicate that the additional financial burden of ESBL infections primarily arises from extended hospital stays, increased diagnostic testing, and higher pharmaceutical costs, rather than direct antibiotic expenditures.

### 3.3. Predictors of Higher Hospital Costs in E. coli Bacteremia

Predictors of higher hospital costs were presented in Table 3. In the GLM analysis, male sex, pneumonia, and time before bacteremia were independently associated with increased bed costs. Male patients had a 38% higher bed cost (CR 1.38, 95% CI 1.07–1.78, *p =* 0.015), while pneumonia increased bed cost by 52% (CR 1.52, 95% CI 1.16–1.99, *p* = 0.002). Each additional day prior bacteremia onset was associated with an 8% increase in bed cost (CR 1.08, 95% CI 1.05–1.10, *p* < 0.001). In contrast, higher CCI was associated with a 40% reduction in bed cost (CR 0.60, 95% CI 0.47–0.77, *p* < 0.001). The model demonstrated good fit (Omnibus *p* < 0.001, deviance/df = 0.826, Pearson χ^2^/df = 0.895).

Diagnostic cost was significantly higher among patients with ESBL *E. coli* bacteremia, pneumonia, and intracranial infection. ESBL infection increased diagnostic cost by 41% (CR 1.41, 95% CI 1.09–1.82, *p =* 0.008), while pneumonia and intracranial infections increased cost by 48% and 34%, respectively. Time before bacteremia also showed a consistent effect, with a 5% increase per day (CR 1.05, 95% CI 1.04–1.06, *p* < 0.001). Conversely, higher CCI was associated with a modest reduction in diagnostic cost (CR 0.86, *p* = 0.029). Model fit indices indicated an acceptable fit.

Pharmacy costs were significantly influenced by infection type and disease severity. Pneumonia and skin and soft tissue infections were associated with increased costs (CR 1.66 and 1.60, respectively), while time before bacteremia increased costs by 6% per day (CR 1.06, *p* < 0.001). The PBS was also associated with increased pharmacy cost (CR 1.07, *p* = 0.005). In contrast, higher CCI reduced pharmacy cost by 36% (CR 0.64, *p* < 0.001). The model demonstrated good fit (deviance/df = 0.788, Pearson χ^2^/df = 0.846).

Total hospital cost was significantly higher among patients with pneumonia (CR 1.55, 95% CI 1.26–1.92, *p* < 0.001) and skin and soft tissue infection (CR 1.34, *p =* 0.033). Time before bacteremia remained a consistent predictor, increasing total cost by 6% per day (CR 1.06, *p* < 0.001). Conversely, higher CCI was associated with a 33% reduction in total cost (CR 0.67, *p* < 0.001). The model demonstrated good fit (deviance/df = 0.502).

Daily cost analysis showed that ESBL *E. coli*, high-care/ICU stay, and PBS were associated with daily expenditure. ESBL infection increases daily cost by 37% (CR 1.37, *p* = 0.026), while high-care/ICU stay was the strongest predictor, increasing daily cost by 116% (CR 2.16, *p* < 0.001). Each point increase in PBS increased daily cost by 5% (CR 1.05, *p* < 0.001). In contrast, time before bacteremia was associated with a 3% decrease in daily cost (CR 0.97, *p* < 0.001). Higher CCI also reduced daily cost (CR 0.76, *p* < 0.001). Model fit was acceptable (deviance/df = 0.291).

Across cost components, time before bacteremia consistently increased cumulative cost (bed, diagnostic, pharmacy, and total cost), while reducing daily cost, suggesting that prolonged hospitalization prior to bacteremia increases overall expenditure but lowers the average daily cost. Pneumonia emerged as a major driver of total, diagnostic, and pharmacy costs. ESBL *E. coli* was specifically associated with increased diagnostic and daily costs. High-care/ICU stay was the strongest predictor of daily cost, while CCI was consistently associated with lower cost estimate across cost components.

### 3.4. Predictors of Higher Hospital Costs in ESBL E. coli Bacteremia

We analyzed predictors of higher hospital costs among 131 patients with ESBL *E. coli* bacteremia (Table 4). We examined factors associated with bed-related costs. The multivariate model for bed cost demonstrated overall statistical significance (*p* < 0.001). Male sex, high-care/ICU stay and time after bacteremia were independently associated with increased bed costs. Male patients had more than double the bed cost (123%) compared with females (CR 2.23, 95% CI 1.69–2.94, *p* < 0.001). High-care/ICU stay increased bed cost by 67% (CR 1.67, 95% CI 1.22–2.28, *p =* 0.001). Each additional day after bacteremia was associated with a 6% increase in bed cost (CR 1.06, 95% CI 1.04–1.08, *p* < 0.001). In contrast, higher comorbidity burden was associated with a 33% reduction in bed cost (CR 0.67, *p* = 0.005). Model showed good fit (deviance/df = 0.700; Pearson χ^2^/df = 0.803).

Diagnostic costs were significantly higher in male patients, pneumonia, high-care/ICU stay, and longer time after bacteremia. Male sex increased diagnostic cost by 30% (CR 1.30, *p* = 0.001), while pneumonia increased cost by 38% (CR 1.38, *p* < 0.001). High-care/ICU stay was associated with a 30% increase (CR 1.30, *p* < 0.001). Each additional day after bacteremia increased diagnostic cost by 4% (CR 1.04, *p* < 0.001). Model fit indices indicated acceptable fit, although deviance/df (0.171) and Pearson χ^2^/df (0.180) suggested mild underdispersion.

Pharmacy cost was influenced by multiple clinical factors. Male sex, pneumonia, skin and soft tissue infection, and high-care/ICU stay were associated with increased pharmacy costs. Male sex increased pharmacy cost by 67% (CR 1.67, *p* < 0.001), while pneumonia and skin/soft tissue infections increased costs by 32% and 58%, respectively. High-care/ICU stay was a strong predictor, increasing pharmacy cost by 85% (CR 1.85, *p* < 0.001). The PBS was also associated with a 7% increase per point (*p =* 0.003), and each additional day after bacteremia was associated with a 6% increase in cost (*p* < 0.001). Conversely, higher CCI reduced pharmacy cost by 24% (CR 0.76, *p* = 0.023). Model fit was acceptable (deviance/df = 0.493).

Total hospital cost was significantly higher among patients with male sex, pneumonia, high-care/ICU stay, and longer time after bacteremia. Male sex increased total cost by 74% (CR 1.74, *p* < 0.001), while pneumonia increased total cost by 33% (CR 1.33, *p* = 0.009). High-care/ICU stay increased total cost by 63% (CR 1.63, *p* < 0.001). Each additional day after bacteremia increased total cost by 5% (CR 1.05, *p* < 0.001). In contrast, higher CCI was associated with a 24% reduction in total cost (CR 0.76, *p =* 0.005). Model fit was acceptable (deviance/df = 0.336).

For daily cost, high-care/ICU stay and PBS were associated with increased daily expenditure, while time after bacteremia and CCI were associated with reduced daily cost. High-care/ICU stay more than doubled daily cost (+118%, CR 2.18, *p* < 0.001), representing the strongest predictor. Each point increase in PBS increased daily cost by 6% (*p* < 0.001). Conversely, each additional day after bacteremia reduced daily cost by 2% (CR 0.98, *p* < 0.001), and higher CCI reduced daily cost by 23% (CR 0.77, *p =* 0.003). Model fit was acceptable (deviance/df = 0.269, Pearson χ^2^/df = 0.306).

Across cost components in ESBL *E. coli* patients, high-care/ICU stay and time after bacteremia were consistent drivers of increased hospital costs, while male sex and pneumonia contributed to higher cumulative costs. In contrast, higher comorbidities were consistently associated with lower cost estimates, and longer time after bacteremia reduced daily cost, suggesting a dilution effect over prolonged hospitalization.

Sensitivity analyses demonstrated a consistent direction of effect for most predictors. ESBL *E. coli*, pneumonia, intracranial infection, and skin/soft tissue infection were associated with increased hospital costs, with effect sizes ranging from 20% to 55% across models. Higher CCI consistently showed a strong inverse association with cost, reducing expenditure by approximately 22–34%. Time before bacteremia and PBS demonstrated a small but stable effect. High-care/ICU stay remained associated with higher costs, although the magnitude varied across models, suggesting potential model sensitivity.

## 4. Discussion

This study demonstrates that hospital costs in *E. coli* bacteremia are influenced by a combination of infection characteristics, healthcare utilization, and patient-related factors. ESBL *E. coli* was consistently associated with higher hospital costs across multiple components, including diagnostic and daily expenditures. This finding reinforces the growing recognition that antimicrobial resistance (AMR) is not only a clinical challenge but also a substantial economic burden, especially in resource-limited healthcare settings. The increased cost of ESBL infections appears to reflect a combination of delayed effective therapy, more complex antimicrobial regimens, and prolonged hospitalization [14,18]. In clinical practice, patients with resistant infections often require escalation to broader-spectrum antibiotics and close monitoring, thereby increasing healthcare utilization [12,19,20]. Recent studies have similarly highlighted that ESBL-producing *Enterobacterales* are associated with longer LOS and higher overall costs, even when adjusting for severity [21,22]. Overall, these findings highlight that ESBL contributes to a multidimensional increase in hospital costs, reinforcing its significance as both a clinical and economic concern. 

The key finding of this study is the substantial impact of high-care/ICU stay on hospital costs. This suggests that the economic burden of bacteremia is largely shaped by illness severity and the need for intensive supportive care [23,24,25]. Recent evidence also shows that ICU-level interventions remain the dominant contributors to hospital expenditure in severe infections [25,26]. ICU care is inherently cost-intensive due to higher staffing ratios, advanced monitoring, invasive procedures, and prolonged LOS, irrespective of the underlying condition [26,27]. Our findings underscore the central role of care intensity in influencing hospital expenditures for bloodstream infections.

The timing of bacteremia onset also showed distinct effects on cost. Recent cohorts of sepsis, HAI, and hospital-onset bacteremia have shown that patients who remain in hospital longer before developing infection accumulate substantially higher total costs and worse outcomes, consistent with more complex underlying conditions and prolonged pre-infection care [28,29,30]. In contrast, in the ESBL subgroup, longer duration after bacteremia increased total cost but reduced daily cost, indicating a dilution effect over prolonged hospitalization. This pattern has been observed in previous economic analyses, where extended LOS increases total cost while reducing average daily expenditure after the early, high-intensity phase of critical illness [31,32,33,34,35]. These results underscore the economic significance of early infection detection, prompt source control, and timely initiation of appropriate therapy, not only to improve clinical outcomes but also to reduce unnecessary downstream expenditures [36,37,38]. A scoping review of source control in sepsis and bloodstream infections concluded that prompt, adequate interventions reduce ICU and hospital LOS and the need for additional procedures, thereby reinforcing economic value [34]. 

Infection source was another important determinant of hospital cost. Pneumonia consistently contributed to higher total, diagnostic, and pharmacy costs, likely reflecting the need for diagnostics, imaging, respiratory support, and broader antimicrobial coverage [39,40]. Similarly, skin and soft tissue infections and intracranial infections were associated with increased costs in specific domains, underscoring that the economic impact of bacteremia varies depending on the underlying source of infection [41,42]. Prior studies have also highlighted pneumonia as one of the most resource-intensive infections due to its association with respiratory failure and ICU utilization [43,44,45].

The inverse association between comorbidity burden and cost deserves careful interpretation. Patients with higher comorbidity index may experience earlier mortality or receive less aggressive interventions, leading to shorter hospital stays and lower cumulative costs [46,47,48,49]. While this may appear paradoxical, similar findings have been reported in recent health economic analyses of severe infections and sepsis [50,51]. Therefore, this finding should not be interpreted as a protective effect but rather as a reflection of healthcare utilization patterns.

Male sex was associated with increased costs, particularly in the ESBL subgroup; however, this association was less consistent in sensitivity analyses, suggesting potential confounding factors. Recent ESBL BSI and UTI studies show that male sex is over-represented among patients with ESBL-producing organisms and recurrent BSIs, which are associated with higher hospital costs. Differences in disease severity, healthcare-seeking behavior, or distribution of infection sources may contribute to this phenomenon, although further investigation is needed [14,52,53].

Disease severity as measured by the PBS was associated with increased pharmacy and daily costs, reinforcing the role of clinical severity in driving resource utilization. Recent studies indicate that PBS more accurately captures acute severity and predicts ICU use and mortality than SOFA in bloodstream infections, suggesting a closer association between higher PBS values and increased hospital costs [54,55]. The PBS also has consistently been established as a predictor of mortality and severe clinical outcomes such as ICU admission, respiratory failure, and renal replacement therapy in bacteremia [56,57]. A retrospective study of community-acquired pyelonephritis caused by ESBL-producing *Enterobacterales* found that each one-point increase in PBS was associated with an additional USD 767 in hospital medical costs, after adjustment for other variables [58]. 

Overall, this study highlights that hospital costs in *E. coli* bacteremia are multifactorial, with high-care/ICU stay, ESBL status, infection source, and timing of infection playing major roles. These findings emphasize the need for targeted strategies to reduce healthcare costs, including early infection control, antimicrobial stewardship, and optimization of critical care utilization [14,59,60]. Addressing these factors may help reduce the economic burden associated with bloodstream infection while improving patient outcomes. 

This study has several limitations. First, its single-center design may limit generalizability to settings with different case-mix, ICU admission thresholds, and cost structures. Second, cost estimates were restricted to direct in-hospital medical costs and did not capture post-discharge or indirect costs, likely underestimating the full economic burden of *E. coli* bacteremia. Although the models adjusted for key clinical and severity-related factors, residual confounding related to institutional and antimicrobial stewardship practices cannot be excluded. Future studies should adopt multicenter and prospective designs and integrate formal cost-effectiveness analyses, including outcomes such as avoided ICU stays, reduced LOS, and quality-adjusted life years. Embedding economic evaluations within antimicrobial stewardship, rapid diagnostic, and infection prevention interventions will be essential to support value-based care strategies and optimize resource allocation in bacteremia management [61,62].

## 5. Conclusions

Hospital costs in *E. coli* bacteremia are driven by infection characteristics, disease severity, and healthcare utilization. ESBL-producing *E. coli* and high-care/ICU stay were major contributors to increased costs, reflecting greater clinical complexity and resource use. The timing of bacteremia and infection source also influenced cost patterns, highlighting differences between cumulative and daily expenditure. Higher comorbidity burden was associated with lower costs, likely reflecting variations in clinical management rather than protective effect. These findings emphasize the importance of antimicrobial stewardship, early infection control, and optimized critical care use to reduce the economic burden of bacteremia. 

## Figures and Tables

**Table 1:** Characteristics of patients with bacteremia caused by *E. coli*.

Variables	Total (*n* = 209)	ESBL *E. coli* (*n* = 131)	Non-ESBL *E. coli* (*n* = 78)	*p*
Demographics				
Sex, male	86 (41.1)	58 (44.3)	28 (35.9)	0.296
Age ≥ 65 years	44 (21.1)	26 (19.8)	18 (23.1)	0.705
Median LOS (IQR)	7 (3–14)	8 (3–16)	6.5 (4–11)	0.237
Median time before bacteremia (IQR)	2 (1–4)	2 (1–6)	1 (1–3)	0.007 *
Median time after bacteremia (IQR)	4 (1–9)	3 (1–10)	4 (2–9)	0.460
Comorbidity				
Hypertension	73 (34.9)	47 (35.9)	26 (33.3)	0.709
Diabetes mellitus	76 (36.4)	51 (38.9)	25 (32.1)	0.317
Heart failure	20 (9.6)	12 (9.2)	8 (10.3)	0.794
COPD	5 (2.4)	3 (2.3)	2 (2.6)	0.901
Liver cirrhosis	12 (5.7)	7 (5.3)	5 (6.4)	0.748
Hematologic malignancy	15 (7.2)	8 (6.2)	7 (9.0)	0.446
Solid tumor	54 (25.8)	32 (24.4)	22 (28.2)	0.546
CCI ≥ 3	121 (57.9)	77 (58.8)	44 (56.4)	0.737
Potential source of bacteremia				
Pneumonia	144 (68.9)	90 (68.7)	54 (69.2)	0.936
Intra-abdominal infection	85 (40.7)	31 (23.7)	54 (69.2)	<0.001 *
Urinary tract infection	102 (48.8)	89 (67.9)	13 (16.7)	<0.001 *
Intracranial infection	60 (28.7)	4 (3.1)	56 (71.8)	<0.001 *
Skin and soft tissue infection	32 (15.3)	32 (24.4)	0 (0)	<0.001 *
Primary bacteremia	7 (3.3)	5 (3.8)	2 (2.6)	0.62
High-care/ICU stay	109 (52.2)	74 (56.5)	35 (44.9)	0.138
HAIs	80 (38.3)	61 (46.6)	19 (24.4)	0.002 *
IIAT	123 (60.6)	109 (83.2)	14 (19.4)	<0.001 *
Mechanical ventilation	75 (35.9)	52 (39.7)	23 (29.5)	0.181
Serum albumin < 30 g/L	166 (79.4)	109 (84.5)	57 (74.0)	0.098
Median SOFA score (IQR)	6 (4–8)	6 (4–8)	5 (2–8)	0.257
Median PBS (IQR)	1 (0–4)	2 (0–4)	0 (0–2)	<0.001 *
Vasopressor use	88 (42.1)	63 (48.1)	23 (32.1)	0.033 *
Mortality	137 (65.6)	91 (69.5)	46 (59.0)	0.164

Note: * = significant at <0.05. Data are expressed as *n* (%) unless otherwise stated. ESBL: extended-spectrum beta-lactamase; IQR: interquartile range; LOS: length of stay; COPD: chronic obstructive pulmonary disease; CCI: Charlson comorbidity index; ICU: intensive care unit; IIAT: Inappropriate initial antibiotic therapy; HAIs: Healthcare-associated infections; SOFA: sequential organ failure assessment; PBS: Pitt bacteremia score.

**Table 2:** Comparison of hospital costs among ESBL *E. coli* and non-ESBL *E. coli* bacteremia (in 2023 USD).

Hospital Cost	ESBL *E. coli* Median (IQR)	Non-ESBL *E. coli* Median (IQR)	*p*
Bed cost	871.48 (383.34–2021.75)	523.14 (278.08–1218.60)	0.009 *
Diagnostic cost	613.55 (424.09–883.21)	408.21 (267.09–608.51)	<0.001 *
Pharmacy cost	869.27 (376.95–1542.28)	483.26 (252.41–1227.65)	0.013 *
Antibiotic cost	18.67 (7.34–43.04)	18.67 (4.08–37.97)	0.358
Total cost	2448.15 (1315.70–4830.98)	1459.16 (1006.08–3324.27)	0.001 *
Daily cost	311.73 (197.30–612.16)	252.88 (157.68–368.90)	0.021 *

Note: * = significant at <0.05. ESBL: extended-spectrum beta-lactamase; IQR: interquartile range.

**Table 3:** Predictors of higher hospital costs in *E. coli* bacteremia.

Predictors	Cost Ratio	CI	*p*
Bed cost			
Male sex	1.38	1.07–1.78	0.015
Pneumonia	1.52	1.16–1.99	0.002
CCI ≥ 3	0.60	0.47–0.77	<0.001
Time before bacteremia	1.08	1.05–1.10	<0.001
Diagnostic cost			
ESBL *E. coli*	1.41	1.09–1.82	0.008
Pneumonia	1.48	1.28–1.71	<0.001
Intracranial infection	1.34	1.07–1.67	0.011
CCI ≥ 3	0.86	0.75–0.98	0.029
Time before bacteremia	1.05	1.04–1.06	<0.001
Pharmacy cost			
CCI ≥ 3	0.64	0.51–0.82	<0.001
Skin infection	1.60	1.15–2.24	0.005
Pneumonia	1.66	1.28–2.15	<0.001
Time before bacteremia	1.06	1.03–1.08	<0.001
PBS	1.07	1.02–1.13	0.005
Daily cost			
ESBL *E. coli*	1.37	1.04–1.81	0.026
CCI ≥ 3	0.76	0.66–0.89	<0.001
High-care/ICU	2.16	1.83–2.54	<0.001
Time before bacteremia	0.97	0.96–0.99	<0.001
PBS	1.05	1.02–1.09	<0.001
Total cost			
Pneumonia	1.55	1.26–1.92	<0.001
Skin and soft tissue infection	1.34	1.02–1.76	0.033
CCI ≥ 3	0.67	0.55–0.81	<0.001
Time before bacteremia	1.06	1.04–1.08	<0.001

Note: CI: confidence interval; CCI: Charlson comorbidity index; ESBL: extended-spectrum beta-lactamase; ICU: intensive care unit; PBS: Pitt bacteremia score

**Table 4:** Predictors of higher hospital costs in ESBL *E. coli* bacteremia.

Predictors	Cost Ratio	CI	*p*
Bed cost			
Male sex	2.23	1.69–2.94	<0.001
CCI ≥ 3	0.67	0.51–0.89	0.005
High-care/ICU	1.67	1.22–2.28	0.001
Time after bacteremia	1.06	1.04–1.08	<0.001
Diagnostic cost			
Male sex	1.30	1.09–1.45	0.001
Pneumonia	1.38	1.19–1.61	<0.001
High-care/ICU	1.30	1.11–1.52	<0.001
Time after bacteremia	1.04	1.03–1.04	<0.001
Pharmacy cost			
Male sex	1.67	1.32–2.10	<0.001
Pneumonia	1.32	1.03–1.71	0.032
Skin and soft tissue infection	1.58	1.13–2.22	0.023
CCI ≥ 3	0.76	0.60–0.96	0.023
High-care/ICU	1.85	1.41–2.42	<0.001
PBS	1.07	1.022–1.11	0.003
Time after bacteremia	1.06	1.04–1.08	<0.001
Daily cost			
CCI ≥ 3	0.77	0.64–0.91	0.003
High-care/ICU	2.18	1.79–2.65	<0.001
PBS	1.06	1.02–1.09	<0.001
Time after bacteremia	0.98	0.98–0.99	<0.001
Total cost			
Male sex	1.74	1.43–2.12	<0.001
Pneumonia	1.33	1.07–1.64	0.009
CCI ≥ 3	0.76	0.62–0.92	0.005
High-care/ICU	1.63	1.31–2.04	<0.001
Time after bacteremia	1.05	1.04–1.07	<0.001

Note: ESBL: extended-spectrum beta-lactamase; CI: confidence interval; ICU: intensive care unit; CCI: Charlson comorbidity index; PBS: Pitt bacteremia score.

## Data Availability

The original data presented in the study are openly available in https://doi.org/10.6084/m9.figshare.31323211.

## Supplementary Materials

The following supporting information can be downloaded at https://www.mdpi.com/article/10.3390/tropicalmed11050116/s1. Table S1: Bivariate analysis among *E. coli* bacteremia based on the median total cost; Table S2: Bivariate analysis among ESBL *E. coli* bacteremia based on the median total cost.

## Abbreviations

The following abbreviations are used in this manuscript:

AIC	Akaike Information Criterion
AIDS	Acquired immunodeficiency syndrome
AMR	Antimicrobial resistance
BIC	Bayesian Information Criterion
BSI	Bloodstream infection
CCI	Charlson comorbidity index
CI	Confidence interval
CR	Cost ratio
ESBL	Extended-spectrum beta-lactamase
GLM	Generalized linear model
HAI	Healthcare-associated infection
IIAT	Initial inappropriate antibiotic therapy
IQR	Interquartile range
PBS	Pitt bacteremia score
qSOFA	Quick sequential organ failure assessment
SIRS	Systemic inflammatory response syndrome
SOFA	Sequential organ failure assessment
SPSS	Statistical product and service solutions
USD	United States Dollar
UTI	Urinary tract infection
