# Supplementary material for: Hospital Cost Components and Predictors in Escherichia coli Bacteremia
Source: Trop Med Infect Dis. 2026 Apr 28;11(5):116. doi: 10.3390/tropicalmed11050116 (PMC13211471; doi:10.3390/tropicalmed11050116)
Supplement: Supplementary file 1 [file tropicalmed-11-00116-s001.zip › tropicalmed-4183484-supplementary.pdf]

Table S1. Bivariate analysis among *E. coli* bacteremia based on the median total cost

Variables	Low-medium cost (*n*=104)	Medium-high cost (*n*=105)	*p*
Demographics			
Male gender, n(%)	34 (32.7)	52 (49.5)	0.013
Age ≥65	24 (23.1)	20 (19.0)	0.475
Median total LOS (IQR)	4 (2-8)	14 (6-19.5)	<0.001
Median time before bacteremia (IQR)	1 (1-2)	3 (1-8)	<0.001
Median time after bacteremia (IQR)	2 (1-5)	8 (2-13)	<0.001
Comorbidity			
Hypertension	31 (29.8)	42 (40)	0.122
Diabetes mellitus	35 (33.7)	41 (39)	0.418
Heart failure	6 (5.8)	14 (13.3)	0.063
COPD	2 (1.9)	3 (2.9)	1.000
Liver cirrhosis	11 (10.6)	1 (1.0)	0.007
Hematologic malignancy	9 (8.7)	6 (5.7)	0.399
Solid tumor	34 (32.7)	20 (19.0)	0.024
Potential source of bacteremia			
Pneumonia	62 (59.6)	82 (78.1)	0.004
Intra-abdominal	40 (38.5)	45 (42.9)	0.518
Urinary tract	44 (42.3)	58 (55.2)	0.062
Intracranial	36 (34.6)	24 (22.9)	0.060
Skin and soft tissue	9 (8.7)	23 (21.9)	0.008
Primary bloodstream infection	6 (5.8)	1 (1.0)	0.121
High-care/ICU stay	**38 (36.5)**	**71 (67.6)**	**<0.001**
HAIs	24 (23.1)	56 (53.3)	<0.001
ESBL *E. coli*	56 (53.8)	75 (71.4)	0.009
CCI ≥3	70 (67.3)	51 (48.6)	0.006
Mechanical ventilation	8 (7.7)	67 (63.8)	<0.001
Serum albumin <30 g/L	80 (79.2)	86 (81.9)	0.625
Median SOFA score (IQR)	5.50 (3.25-7)	6 (4-8)	0.222
Median Pitt score (IQR)	0.5 (0-2)	2 (0-4)	<0.001
Vasopressor use	39 (37.5)	49 (46.7)	0.180
Mortality, n (%)	71 (68.3)	66 (62.9)	0.410

Table S2. Bivariate analysis among ESBL-*E. coli* bacteremia based on the median total cost

Variables	Low-medium cost (*n*=56)	Medium-high cost (*n*=75)	*p*
Demographics			
Male gender, n(%)	19 (33.9)	39 (52.0)	0.039
Age ≥65	12 (21.4)	14 (18.7)	0.695
Median total LOS (IQR)	4.5 (2-8)	14 (6-19)	<0.001

Median time before bacteremia (IQR)	1 (1-2.75)	4 (1-8)	<0.001
Median time after bacteremia (IQR)	1.5 (1-5)	6 (2-13)	<0.001
Comorbidity			
Hypertension	16 (28.6)	31 (41.3)	0.132
Diabetes mellitus	19 (33.9)	32 (42.7)	0.310
Heart failure	2 (3.6)	10 (13.3)	0.055
COPD	1 (1.8)	2 (2.7)	0.739
Liver cirrhosis	6 (10.7)	1 (1.3)	0.018
Hematologic malignancy	5 (9.1)	3 (4.0)	0.233
Solid tumor	18 (32.1)	14 (18.7)	0.076
Potential source of bacteremia			
Pneumonia	33 (58.9)	57 (76.0)	0.037
Intra-abdominal	11 (19.6)	20 (26.7)	0.349
Urinary tract	39 (69.6)	50 (66.7)	0.718
Intracranial	1 (1.8)	3 (4.0)	0.466
Skin and soft tissue	9 (16.1)	23 (30.7)	0.054
Primary bloodstream infection	4 (7.1)	1 (1.3)	0.086
Highcare/ICU stay	22 (39.3)	52 (69.3)	<0.001
HAIs	14 (25.0)	47 (62.7)	<0.001
CCI ≥3	39 (69.6)	38 (50.7)	0.029
Mechanical ventilation	5 (8.9)	47 (62.7)	<0.001
Serum albumin <30 g/L	48 (88.9)	61 (81.3)	0.242
Median SOFA score (IQR)	6 (4-7)	6 (3-8)	0.722
Median Pitt score (IQR)	2 (0-3)	2 (0-6)	0.040
Vasopressor use	23 (41.1)	40 (53.3)	0.165
Mortality, n (%)	40 (71.4)	51 (68.0)	0.673
